# Ethanol Sensors Based on Porous In_2_O_3_ Nanosheet-Assembled Micro-Flowers

**DOI:** 10.3390/s20123353

**Published:** 2020-06-12

**Authors:** Wenbo Qin, Zhenyu Yuan, Hongliang Gao, Fanli Meng

**Affiliations:** College of Information Science and Engineering, Northeastern University, Shenyang 110819, China; 1810318@stu.neu.edu.cn (W.Q.); yuanzhenyu@ise.neu.edu.cn (Z.Y.); gaohongliang@ise.neu.edu.cn (H.G.)

**Keywords:** In_2_O_3_, nanosheet-assembled micro-flowers, hydrothermal method, ethanol, gas sensors

## Abstract

By controlling the hydrothermal time, porous In_2_O_3_ nanosheet-assembled micro-flowers were successfully synthesized by a one-step method. The crystal structure, microstructure, and internal structure of the prepared samples were represented by an x-ray structure diffractometry, scanning electron microscopy, and transmission electron microscopy, respectively. The characterization results showed that when the hydrothermal time was 8 h, the In_2_O_3_ nano materials presented a flower-like structure assembled by In_2_O_3_ porous nanosheets. After successfully preparing the In_2_O_3_ gas sensor, the gas sensing was fully studied. The results show that the In_2_O_3_ gas sensor had an excellent gas sensing response to ethanol, and the material prepared under 8 h hydrothermal conditions had the best gas sensing property. At the optimum working temperature of 270 °C, the highest response value could reach 66, with a response time of 12.4 s and recovery time of 10.4 s, respectively. In addition, the prepared In_2_O_3_ gas sensor had a wide detection range for ethanol concentration, and still had obvious response for 500 ppb ethanol. Furthermore, the gas sensing mechanism of In_2_O_3_ micro-flowers was also studied in detail.

## 1. Introduction

With the rapid development of social industry, the problem of gas pollution has become more and more serious. Pollution gases from all sides such as car exhausts of toxic and harmful gas, methane gas in mine methane gas leaks, formaldehyde from interior decorations and new car interiors, and toxic gas from industrial emissions not only seriously affect the quality of the environment, but also cause great harm to the human body. Therefore, fast and exact detection of poisonous and harmful gases has become very urgent [1,2]. Sensors, just like human organs, can sense changes in the outside world and transmit the obtained information [3,4]. Gas sensors like electronic noses can detect and monitor gas to prevent safety accidents [5]. In recent decades, in order to obtain better gas sensitivity, researchers have made a variety of gas sensors such as semiconductor sensors [6,7], infrared sensors [8], electrochemical sensors [9], thermal conductivity sensors [10], solid electrolyte sensors [11], and so on. Among them, gas sensors made by semiconductors, especially metal oxide semiconductors (MOS), have attracted extensive attention due to their advantages of environmental protection, simple preparation, low cost, and good stability [12,13,14]. Among a large number of MOS materials, In_2_O_3_, as a typical n-type semiconductor material, has become a hot research topic due to its wide bandgap width and low resistivity, and has been widely used in liquid crystal devices, solar cells, and gas sensors [15,16,17]. By referring to the previous research results, it has been found that In_2_O_3_ nanomaterials show good gas sensing response to both oxidation and reductive gases, so In_2_O_3_ is a gas-sensitive material with great research potential.

The research objects of gas sensors are mostly toxic and harmful gases such as volatile organic compound (VOC) gases, which is very harmful to human health [18,19]. In severe cases, people will suffer from convulsions and coma, which will harm the liver, kidney, brain, and nervous system, causing serious consequences such as memory loss [20]. Ethanol, commonly known as alcohol, is the most common VOC gas and an important material in national defense, agriculture, industry, and other fields. In addition, ethanol is also an important detection object for traffic police in the process of law enforcement, so the preparation of high-performing ethanol gas sensors is also a key part of preventing social hazards [21]. Therefore, quickly and precisely detecting ethanol is very important [22,23]. In recent years, researchers have prepared a large number of MOS gas sensors around ethanol gas including ZnO gas sensors [24], Fe_2_O_3_ gas sensors [25], In_2_O_3_ gas sensors [26], SnO_2_ gas sensors [27], and so on. However, a traditional single MOS gas sensor has certain limitations such as high detection threshold, poor selectivity, low response value, high operating temperature, etc., which limit the application of a MOS gas sensor in a real environment [28,29]. Therefore, a variety of research schemes for improving MOS gas sensors have been proposed including noble metal modification [30], construction of heterojunction [31], optimizing microstructure [32], etc. In these schemes, optimizing microstructure does not need the addition of other materials, and porous nanomaterials with high specific surface area are only prepared by changing the reaction conditions or precursors, which is currently a hot research topic. Among them, three-dimensional (3D) layered structures have attracted extensive attention from scholars due to their novel physical properties, porous nanostructures, and large surface area [33,34]. Therefore, porous In_2_O_3_ nanosheet-assembled micro-flowers with high specific surface area are helpful to obtain high performance ethanol gas sensors.

Here, we synthesized the 3D porous flower-like structure assembled by In_2_O_3_ nanosheets by the one-step hydrothermal method. By controlling the time of the hydrothermal reaction (0.5 h, 2 h, 4 h, 6 h, 8 h, 10 h), the formation mechanism was studied. After representing the microstructure of the prepared samples by various characterization methods, the results showed that the perfect In_2_O_3_ flower-like porous structure was successfully obtained under the condition of a 8 h reaction. Then, we prepared a gas sensor and its gas sensitive properties were studied. The results showed that the gas sensor for ethanol had excellent gas-sensing properties, in particular, the 3D In_2_O_3_ flower porous material prepared under 8 h hydrothermal conditions had the best responses of ethanol and good selectivity, stability, and fast response and recovery time. In addition, the gas-sensitive mechanism of 3D In_2_O_3_ flower-like porous material to ethanol was studied in detail.

## 2. Materials and Methods

### 2.1. Materials

All the reagents including indium nitrate (In(NO_3_)_3_·5H_2_O), urea (CN_2_H_4_O), polyvinyl piroxanone (PVP), and anhydrous ethanol (C_2_H_6_O) came from Shandong Xiya reagent company. All reagents were analytically pure and needed no further treatment. Distilled water was used in the whole experiment.

### 2.2. Synthesis of Porous In_2_O_3_ Nanosheet-Assembled Micro-Flowers

Porous In_2_O_3_ nanosheet-assembled micro-flowers were synthesized by a one-step hydrothermal process followed by heat treatment. A total of 1.2 g of indium nitrate and 0.72 g of urea were dissolved in 40 mL of deionized water and stirred for 30 min until fully dissolved. Then, 0.8 g PVP was added to the mixture solution and stirred vigorously until the mixture solution was clear and free of suspension. The obtained clarified precursor solution was transferred to two 30 mL reactors and placed in the oven for hydrothermal reaction at 120 °C for 8 h. After the hydrothermal reaction was completed and the reactor was naturally cooled to room temperature, the reactants obtained were centrifuged with anhydrous ethanol and deionized water several times to remove impurities. The solid precipitate was placed in an oven at 60 °C for drying for 12 h. Finally, the dried product was ground into powder with an abrasive body and transferred to the magnetic boat. Then, the product was placed in a muffle furnace at 600 °C for 2 h with the heating rate of 2 °C/min. The obtained porous In_2_O_3_ nanosheet-assembled micro-flowers were stored in a dry place for preservation. Although the other conditions were unchanged, we changed the hydrothermal reaction times (0.5 h, 2 h, 4 h, 6 h, 10 h) to obtain the comparison test materials, which were recorded as In_2_O_3_-x h (x = 0.5, 2, 4, 6, 8, 10).

### 2.3. Characterization

The microstructure of the obtained In_2_O_3_-x h samples materials were observed by scanning electron microscopy (SEM), and the element content of the material was analyzed by an energy dispersive spectrometer (EDS), model JSM-7001, with a resolution of 1.2 nm, produced by Nippon electronics Co. Ltd in Guangzhou, China. The crystal structure of In_2_O_3_-x h was undertaken with x-ray diffraction (XRD) using the model X’pert pro MRD, manufactured by PANalytical B.V. The internal structure of the obtained sample was observed by transmission electron microscopy (TEM) with model JEM2100PLUS, which was manufactured by JEOL (BEIJING) Co., Ltd. in Beijing, China. The point resolution was 0.23 nm and the line resolution was 0.14 nm.

### 2.4. Gas Test System

**Figure 1:**
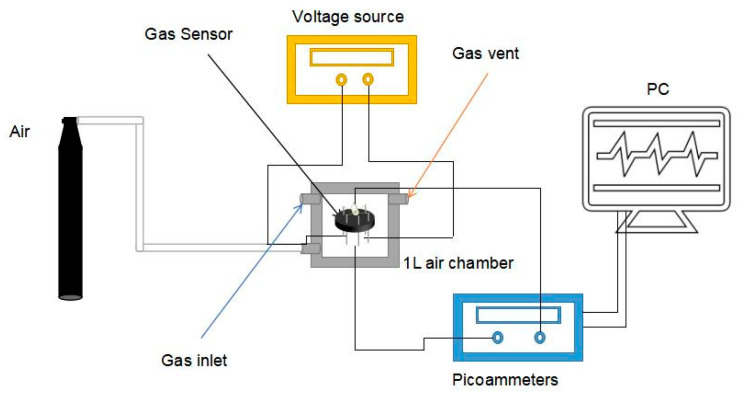
Gas testing unit.

The construction of the gas detection system and the preparation of the gas sensor have been described in detail in our previous work [35], as shown in Figure 1. First, to prepare the In_2_O_3_-x h gas sensor, a small amount of the In_2_O_3_-x h sample material needs to be dispersed in a certain amount of anhydrous ethanol. In order to ensure the uniform dispersion of the In_2_O_3_-x h material, ultrasonic treatment can be carried out in the ultrasonic dispersion instrument for several seconds. Then, the In_2_O_3_-x h suspension is absorbed with a micro pipette and daubed on the surface of the prepared ceramic tube. The In_2_O_3_-x h gas sensor can be obtained by rotating the ceramic tube until the surface material is even by using the rotating smear technology. The prepared In_2_O_3_-x h gas sensor is placed in a 1L closed air chamber with a data receiving base, and the picoammeter, adjustable power supply, and computer terminal are connected. During the gas test, the loading voltage at both ends of the gas sensor can be adjusted by the adjustable power supply. Different voltages can make the nickel complex gold wire in the ceramic tube produce different temperatures, ensuring the control of the working temperature of the gas sensor. The picoammeter is the data receiving end of the gas sensor, and the receiving current can reach the picoammeter level, which can detect extremely small changes in the current. In the LABVIEW program, a virtual voltage source (1 V) is used to calculate the resistance change and save the data. There are two air holes in the airtight chamber. One is the air inlet, which is responsible for the input of target gas. The other is the vent hole, which can discharge the detected gas and make the sensor reach the initial stable state again. According to the saturated vapor pressure of the gas, the specific volume value of the corresponding test gas was calculated and absorbed through the syringe in the laboratory. The injector, containing a certain amount of test gas, will slowly inject the gas into the air chamber through the injection hole. Due to the strong volatilization of VOC gas, the test gas can quickly fill the air chamber. When the change of sensor resistance is completely stable, it means that the sensor test is over. By opening the air cylinder valve, the synthetic air will quickly pass through the chamber and carry away the test gas. The sensor in the air environment will be stabilized again and used for the next measurement. The response value (S) of the n-type gas sensor can be expressed by the change of resistance:

(1) $$S=\frac{R_{a}}{R_{g}}$$

where $R_{a}$ is the resistance value when the gas sensor is stabilized in the air and $R_{g}$ is the resistance value when the gas sensor is stabilized in the target gas. In addition, the response time is generally defined as the time required from the beginning of the reaction to the resistance reaching 90% stable resistance, and correspondingly, the recovery time is the time when the sensor resistance value changes to 90% stable state from the beginning of blowing.

## 3. Results and Discussions

### 3.1. Characterization

**Figure 2:**
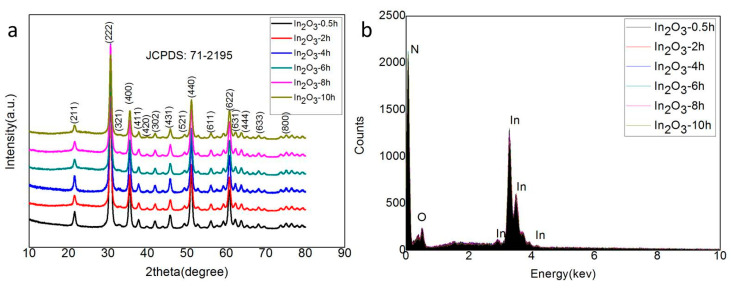
(**a**) X-ray diffraction (XRD) pattern of the samples; (**b**) Energy dispersive (EDS) spectrum analysis of the samples.

The In_2_O_3_-x h nanomaterial was synthesized by the simple one-step hydrothermal method, and its crystal structure was characterized by an x-ray diffractometer. The XRD results of the In_2_O_3_-0.5 h, In_2_O_3_-2 h, In_2_O_3_-4 h, In_2_O_3_-6 h, In_2_O_3_-8 h, and In_2_O_3_-10 h materials are shown in Figure 2a. The results show that all samples had similar diffraction peaks. Compared with the standard JCPDS (Joint Committee on Powder Diffraction Standards) card, it was found that all the diffraction peaks (211), (222), (321), (400), (411), (420), (302), (431), (521), (440), (611), (622), (631), (444), (633), and (800) could correspond to the diffraction peaks of No. 71-2195 cubic indium oxide. The absence of other impurity characteristic peaks indicates that the purity of the synthesized indium oxide was very high. In addition, the Scherrer formula can be used to estimate the grain size [36]:

(2) D=Kλ/Bcosθ

where K is the Scherrer constant (K = 0.943 for cubic particles); B can be the half height and width of the diffraction peak; (λ = 0.154056 nm) is the x-ray wavelength; and θ is the Bragg diffraction angle. According to the XRD diffraction data, the grain size of the In_2_O_3_-8 h sample can be estimated as 17.5 nm. In order to further study the element composition of the composite material, EDS was used to conduct qualitative and quantitative analysis of the elements contained in the material. The results are shown in Figure 2b. It is easy to see that regardless of the hydrothermal time, all synthetic materials only contained In and O elements, and the ratio of their contents was about 2:3, which also indicates the successful preparation of the In_2_O_3_ material. As for the high content of element N, it may be attributed to the inadequate decomposition of excessive urea.

**Figure 3:**
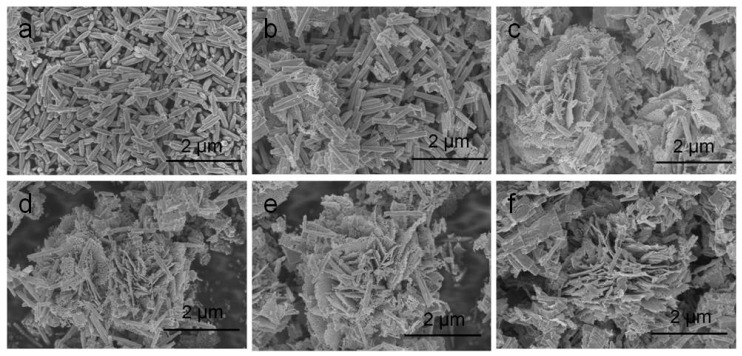
Scanning electron microscopy (SEM) microstructure diagram of the sample: (**a**) In_2_O_3_-0.5 h; (**b**) In_2_O_3_-2 h; (**c**) In_2_O_3_-4 h; (**d**) In_2_O_3_-6 h; (**e**) In_2_O_3_-8 h; (**f**) In_2_O_3_-10 h.

**Figure 4:**
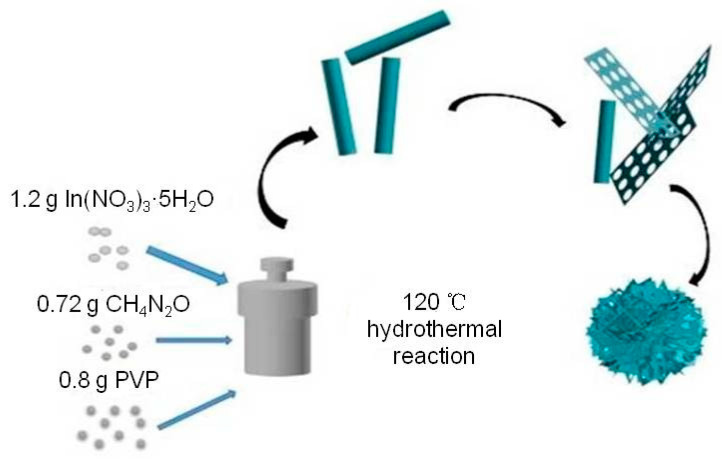
In_2_O_3_ flower structure formation mechanism diagram.

A typical low-power SEM image of the In_2_O_3_-x h nanomaterials is shown in Figure 3, where the length and diameter of the In_2_O_3_-0.5 h nanorods were about 500 nm and 100 nm, respectively. When the hydrothermal time increased to 2 h, the porous In_2_O_3_ nanometer chip began to appear. The thickness of the nanometer chip was about 3 nm. At this time, In_2_O_3_-2 h is the coexistent state of nanorods and nanosheets. With the increase in hydrothermal time, In_2_O_3_-4 h, In_2_O_3_-6 h, and In_2_O_3_-8 h also presented the co-existence of nanorods and nanosheets. When the hydrothermal time increased to 10 h, the In_2_O_3_ nanosheets and nanorods gradually disappeared, and the block structure occupied the main position. Interestingly, when the hydrothermal time was 8 h, the nanorods were assembled into flower-like structures, while the nanorods acted as bridges to connect the scattered petals. Therefore, the formation mechanism of flower-shaped In_2_O_3_ nanomaterials can be visualized in Figure 4.

**Figure 5:**
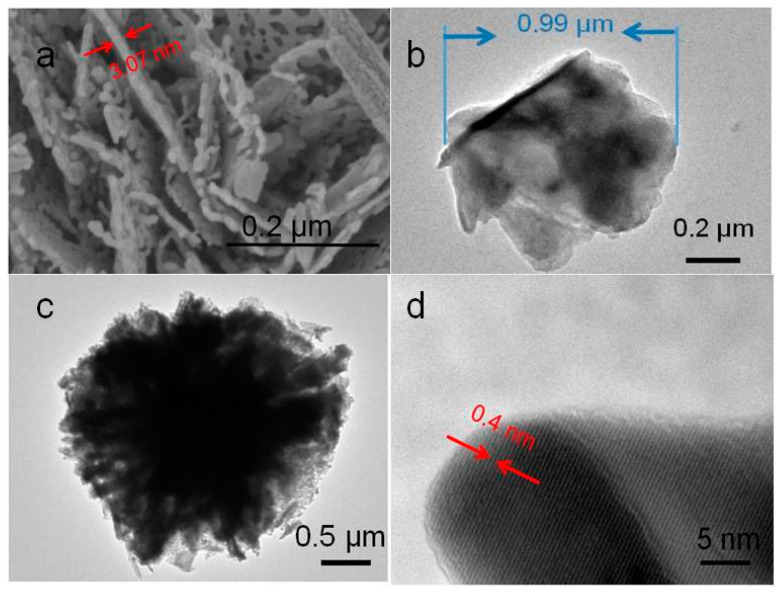
(**a**) High resolution SEM and (**b**–**d**) transmission electron microscopy (TEM) images of In_2_O_3_-8 h.

In order to further study the internal structure of In_2_O_3_ micro-flowers, high-resolution transmission electron microscopy was used to characterize the In_2_O_3_-8 h materials, as shown in Figure 5. The In_2_O_3_ nanosheets presented an irregular shape, with diameter of about 0.99 µm and the pieces stacked together. It can be easily seen in Figure 5c that In_2_O_3_-8 h presented a 360-degree radial shape, and the nanosheets assembled together, finally forming a flower-like structure. Figure 5d shows the high-resolution transmission characterization diagram of the In_2_O_3_-8 h micro-flowers. It can be found that the In_2_O_3_-8 h micro-flowers presented a good crystalline state, with the crystal plane spacing of about 0.4 nm, which was attributed to the (211) crystal plane of the In_2_O_3_ cubic phase. All these characterization results of the In_2_O_3_-8 h samples indicate the successful synthesis of the three-dimensional structure of In_2_O_3_ micro-flowers.

### 3.2. Gas Sensitivity Test of the In_2_O_3_ Gas Sensor

Based on the importance and urgency of ethanol gas detection, we systematically studied the gas sensing characteristics of the In_2_O_3_-x h (x = 0.5, 2, 4, 6, 8, 10) gas sensors with ethanol as the main research object. As is widely known, for gas sensors, the determination of the optimal operating temperature is the basis of the gas sensor for gas sensing research. When the operating temperature is low, the activity of gas molecules is low, and less gas is adsorbed on the surface of the sensitive material, resulting in a low response value of the gas sensor. As the working temperature gradually increases, a large number of gas molecules gain enough energy to stimulate the activity, and the number of gas molecules adsorbed on the surface of the sensitive material increases, resulting in improved response. However, when the temperature continues to increase and exceeds a certain value, the gas molecules will be overactive and it will be difficult to adsorb on the adsorption site of the material surface, which will lead to the performance decline of the gas sensor. This critical temperature value is generally defined as the best operating temperature of the gas sensor.

**Figure 6:**
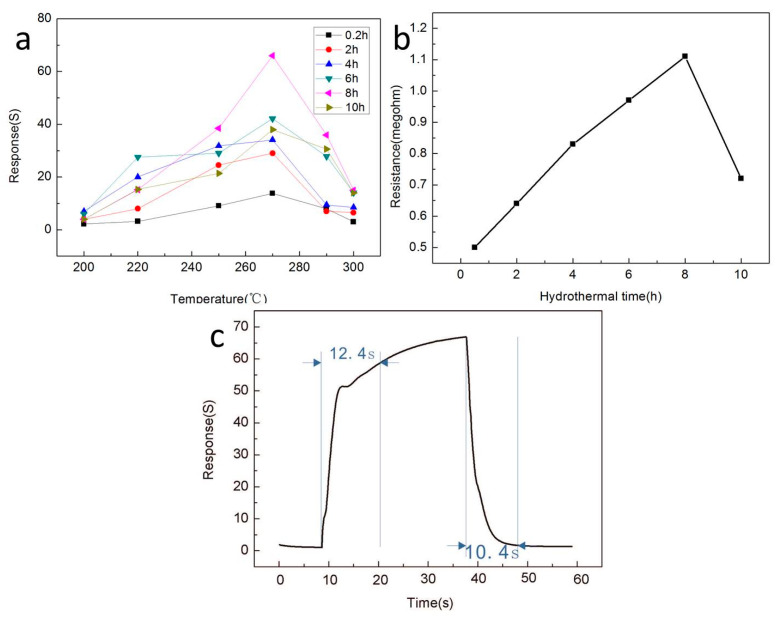
(**a**) The response curves of different sensors to 100 ppm ethanol gas at different operating temperatures; (**b**) Resistance values in air of the nanostructured sensors prepared under different hydrothermal action times; (**c**) Response curve of the In_2_O_3_-8 h sensor to 100 ppm ethanol at the optimum operating temperature of 270 °C.

In order to determine the optimal operating temperature of the In_2_O_3_-x h gas sensor, we set the operating temperature range of 200–300 °C to conduct the gas sensing test on the prepared sensor. Ethanol was the only target gas, and the results are shown in Figure 6a. It is easy to observe that the In_2_O_3_-x h gas sensor presented a typical volcanic variation trend, and the optimum operating temperature of the In_2_O_3_-x h gas sensor could be determined as 270 °C. All subsequent gas sensing tests were performed at 270 °C. In addition, it was also easy to see that the In_2_O_3_-8 h sensor had the best response to 100 ppm ethanol gas at the optimal operating temperature, which can be attributed to the excellent flower-like porous structure of In_2_O_3_-8 h, causing absorption of more ethanol molecules compared with other materials. The resistance values of the In_2_O_3_ gas sensors prepared under different hydrothermal conditions in air are shown in Figure 6b, where the voltage source was fixed at 1 V. It is obvious that the resistance value of the gas sensor varies greatly with the difference in the hydrothermal time, and it generally tends to increase first and then decrease, which can be explained by the microstructure of the material. When the hydrothermal reaction time is short, In_2_O_3_ presents a rod-like structure, which is densely packed together and in full contact, so the resistance of the materials is low. With the increase in hydrothermal time, a porous lamellar structure appears, which leads to the increase in the oxygen adsorption potential, the increase in electron transfer, and the increase in material resistance. Second, the incomplete contact accumulation of the sheet structure will also increase the resistance value. When the hydrothermal time continued to increase and exceeded 10 h, the material began to present a blocky structure, with a decrease in oxygen adsorption potential and electron transfer, leading to a decrease in resistance value. Throughout the whole process, it can be found that when the hydrothermal time was 8 h, In_2_O_3_ had the most oxygen adsorption potential, the largest electron transfer, the largest electrical resistance, and the best gas-sensitive characteristics. On this basis, we selected the In_2_O_3_-8 h gas sensor as the optimal gas sensor, and first studied its response time and recovery time, which is shown in Figure 6c. The results showed that the response time of the In_2_O_3_-8 h sensor was 12.4 s and the recovery time was 10.4 s for 100 ppm ethanol. We compared the ethanol gas sensitivity of the prepared In_2_O_3_-8 h gas sensor with previous studies, and the results are shown in Table 1. In contrast, the In_2_O_3_-8 h gas sensor prepared by us still had excellent gas sensing.

**Figure 7:**
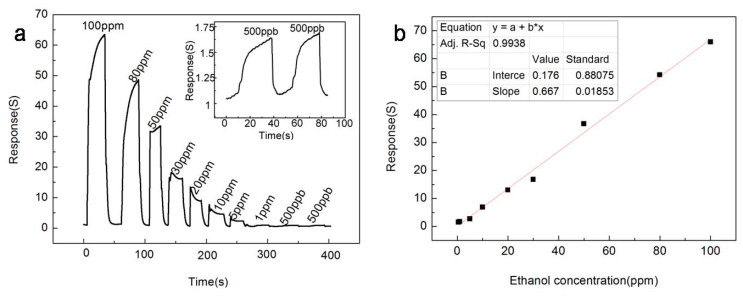
At the optimum operating temperature of 270 °C, (**a**) the response curve of the In_2_O_3_-8 h sensor to ethanol gas at different concentrations; (**b**) The relationship between the In_2_O_3_-8 h sensor response value and ethanol concentration.

Figure 7 shows the gas sensing response of the In_2_O_3_-8 h gas sensor to ethanol of different concentrations at the optimal operating temperature of 270 °C. Obviously, as the concentration of ethanol increased, the response of In_2_O_3_-8 h gas sensor also increased, and when ethanol gas was released, the In_2_O_3_-8 h gas sensor could return to the initial equilibrium state, which indicates that the In_2_O_3_-8 h gas sensor has excellent continuity and recovery performance. In addition, from Figure 7a, we can easily observe that the In_2_O_3_-8 h gas sensor still had significant response changes for the ethanol gas of 500 ppb, and that the same stable state could be maintained for two consecutive measurements, indicating that the detection limit of the In_2_O_3_-8 h gas sensor could reach the level of ppb. Figure 7b shows the fitting curve between the response value of the In_2_O_3_-8 h gas sensor and the concentration of ethanol gas, where is easy to see that there is a good linear relationship between the response value and concentration, indicating that the In_2_O_3_-8 h gas sensor has a wide detection range.

**Figure 8:**
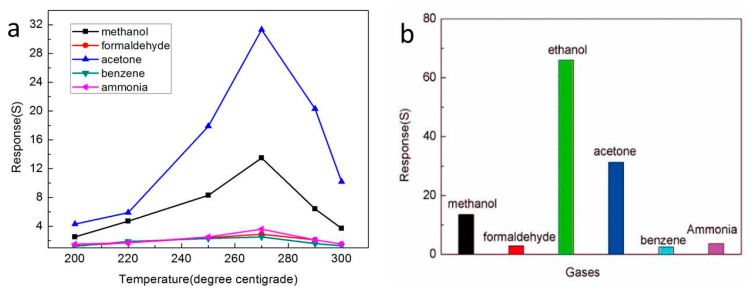
(**a**) The gas-sensitive characteristics of different sensors for different gases; (**b**) The gas sensing response of the In_2_O_3_-8 h sensor to different gases.

Selectivity is an important property of gas sensors. To study the gas sensing characteristics of the In_2_O_3_-8 h gas sensor to different gases, we particularly selected 100 ppm of methanol, formaldehyde, acetone, benzene, and ammonia as the comparison, and tested the response value of the In_2_O_3_-8 h gas sensor to different gases at different working temperatures up to 270 °C, as shown in Figure 8a. Obviously, at the 270 °C operating temperature, the In_2_O_3_-8 h sensor had the best gas sensitivity to different gases. Therefore, 270 °C was selected as the optimal operating temperature and the selectivity of the sensor to different gases was studied. The results are shown in Figure 8b where the response value of the In_2_O_3_-8 h gas sensor to the other five gases was very low, and its response to ethanol gas was at least twice that of the other five gases, indicating that the In_2_O_3_-8 h gas sensor has good selectivity.

**Figure 9:**
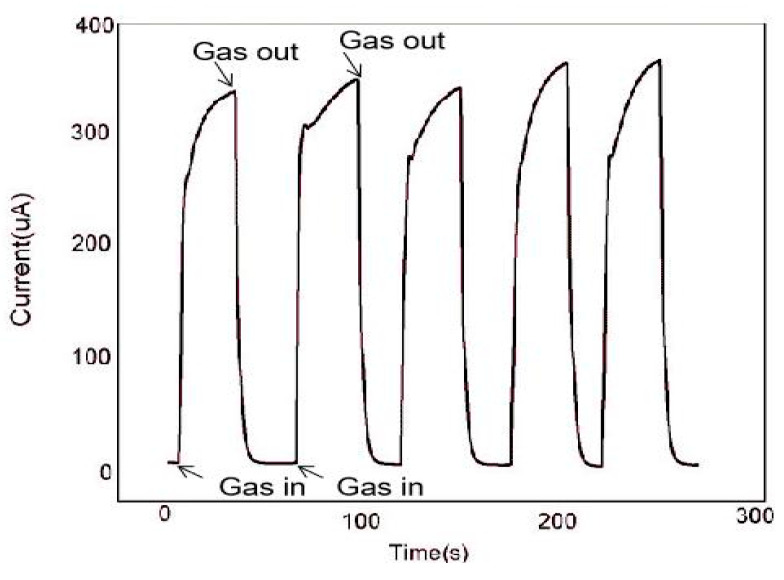
At 270 °C, five consecutive measurements of the In_2_O_3_-8 h sensor for 100 ppm ethanol.

**Figure 10:**
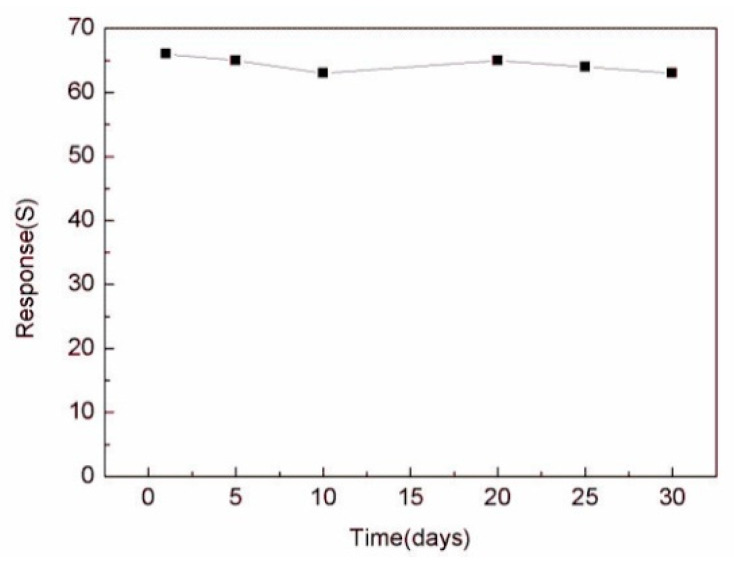
The stability of the In_2_O_3_-8 h sensor was tested for the duration of one month.

The continuity of the gas sensor is also an important research index, which is an important standard to detect whether the gas sensor can measure continuously. In Figure 9, we used the In_2_O_3_-8 h gas sensor to make five consecutive measurements of 100 ppm ethanol gas at the operating temperature of 270 °C. The results show that after each inlet and outlet cycle, the In_2_O_3_-8 h gas sensor could return to the initial equilibrium state, similar to the previous one. By comparing the measured values of the five cycles, the error range of the sensor was found to be between 2.9% and 5%, which shows that the sensor maintained robust response performance. In addition, we also tested the In_2_O_3_-8 h gas sensor repeatedly for a month under the same conditions. Figure 10 shows the In_2_O_3_-8 h gas sensor with six tests of 100 ppm ethanol gas in one month. The results showed that the response value did not change significantly, and the average value remained around 65, indicating its excellent stability.

### 3.3. Gas Sensing Mechanism of the In_2_O_3_-8 h Gas Sensor

**Figure 11:**
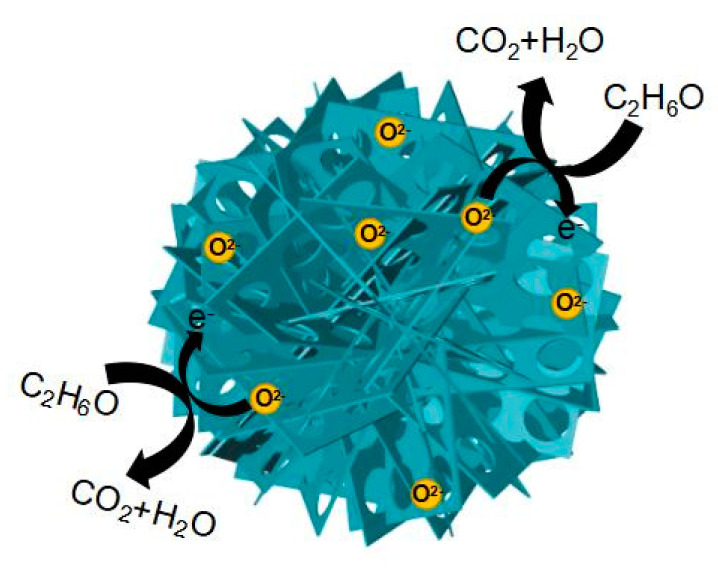
The mechanism of ethanol gas sensing of the In_2_O_3_-8 h micro-flowers.

The gas sensing mechanism of the In_2_O_3_-8 h gas sensor is mainly due to the redox reaction of ethanol molecules and adsorbed oxygen on its surface [47,48]. When metal oxide semiconductor sensors work, oxygen molecules in the air are adsorbed on the surface of sensitive materials and produce adsorbed oxygen. At this point, the sensitive material will release electrons to the adsorbed oxygen, which will form oxygen anions to attach to the surface of the sensitive material and form an ion layer, namely an electron depletion layer. When the ethanol molecules come into contact with the sensitive material, the ethanol molecules react with the oxygen anions in a redox reaction, releasing electrons back to the sensitive material at the same time, which results in a decrease in the thickness of the electron depletion layer and a sharp decrease in the resistance value of the gas sensor, as shown in Figure 11. When ethanol gas is released, the In_2_O_3_-8 h gas sensor will exchange electrons with the adsorbed oxygen again, and the thickness of the electron depletion layer will increase accordingly, and the resistance value of the sensor will return to its initial state. The oxygen anion has three forms, namely O_2_^−^, O^−^, and O^2−^, whose state is determined by the operating temperature. Generally, when the operating temperature is lower than 150 °C, the surface of MOS is mainly O_2_^−^. When the temperature rises but not above 200 °C, O_2_^−^ will gradually change to O^−^; when the operating temperature is above 200 °C, O^2−^ is the main constituent. Therefore, the sensitivity mechanism of the In_2_O_3_-8 h gas sensor to ethanol gas is as follows:

(3) $$O^{2}\left(g\right)=O^{2}\left(\mathrm{ad}\right)$$

(4) $$O^{2}\left(\mathrm{ad}\right)+2e^{-}=O^{2-}\left(\mathrm{ad}\right)$$

(5) $$7O^{2-}\left(\mathrm{ad}\right)+C_{2}H_{6}O=2H_{2}O+3{\mathrm{CO}}_{2}+14e^{-}$$

The excellent gas sensing of the sensor can be attributed to two aspects: The surface effect brought by the small size of nanomaterials and the unique 3D microstructure, as we know that the surface effects of small nanomaterials include an increase in surface area and an increase in the number of atoms on the surface. This results in a very high surface energy and atomic coordination deficit, which makes the surface atoms very reactive and easy to combine with other atoms. The thickness of the prepared nanosheet is about 3 nm, and its small size effect will lead to a large surface area and promote the combination of ethanol molecules and sensitive materials to obtain high gas sensing. In addition, the excellent gas sensing of the In_2_O_3_-8 h gas sensor is mainly attributed to its unique nanostructure, namely porous In_2_O_3_ nanosheet-assembled micro-flowers [49,50], as shown in Figure 11. On one hand, the flower-like structure of In_2_O_3_-8 h can provide more oxygen molecular adsorption sites to produce more adsorbed oxygen, and the large amount of oxygen negative ions generated at the same time can thicken the electron depletion layer, resulting in an increase in the resistance of the gas sensor. Therefore, when the ethanol molecule is in contact with the electron depletion layer on the surface of the sensitive material and the redox reaction occurs, a larger change in the resistance value can be generated, and a larger response value can be generated when the reaction occurs in the sensor’s gas sensing. On the other hand, the existence of a porous structure can facilitate the gas molecules to enter the inside of the sensitive material, which is conducive to the dispersion of the gas molecules in the sensitive material. Gas molecules can redox with more adsorbed oxygen quickly, and when the reaction is finished, gas molecules can also leave quickly, which ensures that the In_2_O_3_-8 h gas sensor has the characteristics of rapid response and recovery. In conclusion, the micro-flower structure of In_2_O_3_-8 h promotes its rapid and efficient detection of ethanol gas.

## 4. Conclusions

In this paper, we successfully synthesized porous In_2_O_3_ nanosheets through the one-step hydrothermal method. The porous In_2_O_3_ nanosheet-assembled micro-flowers were prepared by controlling the hydrothermal reaction time, and the synthesis mechanism was also studied. In_2_O_3_ material was characterized by various characterization methods, and the successful synthesis of the In_2_O_3_ cubic phase and micro-flower structure was proven. We successfully prepared the In_2_O_3_ gas sensor and studied its ethanol detection performance. The results show that the In_2_O_3_-8 h gas sensor can detect ethanol gas quickly and efficiently, and the highest response to 100 ppm ethanol could reach 66 at 270 °C. The response time and recovery time were only 12.4 s and 10.4 s. In addition, the In_2_O_3_-8 h gas sensor also has excellent continuity, stability, and selectivity, and the detection limit reached the level of hundreds of ppb, which is a potential material for the detection of ethanol gas.

## Figures and Tables

**Table 1:** The gas sensitivity of various ethanol sensing materials to ethanol in current studies.

Sensing Material	Concentration (ppm)	Temperature (°C)	Response (Ra/Rg)	Response Time (s)/Recovery Time (s)	Detection Limits	Ref.
Fe-doped In_2_O_3_ nanospheres	100	350	133	15/55	5 ppm	[37]
TeO_2_/In_2_O_3_ nanorods	50	300	2.28	160/200	50 ppm	[38]
In_2_O_3_ nanofibers	100	300	14.3	2/5	1 ppm	[39]
In_2_O_3_ nanostructures	100	320	20	11/4	20 ppm	[40]
Cr_2_O_3_/ZnS nanorods	200	300	13.84	23/20	5 ppm	[41]
In_2_O_3_/ZnS rough microspheres	100	260	11.7	21/34	10 ppm	[42]
In_2_O_3_ hollow nanorod	100	200	38.6	8/6	5 ppm	[43]
Flower-like In_2_O_3_	100	320	27.6	18/9	2 ppm	[44]
LaNi_1-x_Ti_x_O_3_ nanoparticles	200	300	0.5	24/44	50 ppm	[45]
Pd-nanoparticles decorated ZnO-nanorod	500	260	5.12	6/95	100 ppm	[46]
In_2_O_3_ nanoflower	100	270	66	12.4/10.4	0.5 ppm	This work
